# Defining kratom: evidence-based policy guidelines for a rapidly evolving market

**DOI:** 10.3389/fphar.2026.1925809

**Published:** 2026-10-22

**Authors:** Erin Berthold, Paula N. Brown, Michael Chan, Oliver Grundmann, Christopher R. McCurdy, Abhisheak Sharma, Darshan Singh

**Affiliations:** 1 Planted in Science Consulting, LLC, St Augustine, FL, United States; 2 Natural Health & Food Products Research Group, Centre for Applied Research & Innovation, BC Institute of Technology, Burnaby, BC, Canada; 3 Pharmacognosy Institute and Department of Pharmaceutical Sciences, Retzky College of Pharmacy, University of Illinois Chicago, Chicago, IL, United States; 4 Department of Medicinal Chemistry, College of Pharmacy, University of Florida, Gainesville, FL, United States; 5 Department of Pharmaceutics, College of Pharmacy, University of Florida, Gainesville, FL, United States; 6 Centre for Drug Research, Universiti Sains Malaysia, Penang, Malaysia

**Keywords:** cGMP, indole alkaloid, kratom, mitragynine, supplement

## Abstract

The leaves of *Mitragyna speciosa,* commonly called kratom, have been consumed for centuries in Southeast Asia for their energizing and analgesic properties. Over the last decade kratom has gained popularity in the United States, with an estimated 10–16 million consumers, and a wide variety of products have become available in the marketplace. Along with a plethora of product formulations that contain various concentrations of kratom alkaloids, new products have emerged, labelled as “kratom”, but comprised of highly concentrated synthetic alkaloids. Consumers and regulators alike struggle to differentiate the spectrum of products that range from those similar in composition to traditional kratom to those that are illegally marketed, unapproved novel drugs. With significant implications to public health policy and safety, there is a need to clearly define and differentiate kratom, kratom-derived, and synthetic products. Herein we offer guidance to support standardization of the terminology used in the manufacture and labelling of kratom and kratom-derived products in the United States. This is intended to be a reference tool for use by industry and federal, state, and other jurisdictions that oversee the kratom industry to encourage clear and consistent communication.

## 1 Introduction

Kratom [*Mitragyna speciosa* (Korth.) Havil.] is a tree with a long history of use in Southeast Asia where the fresh leaves are chewed or brewed in water and consumed primarily as a restorative to improve focus, increase energy and endurance, and alleviate minor aches and pains from physical labor (Suwanlert, 1975; Babu et al., 2008; Vicknasingam et al., 2010; Tanguay, 2011; Barceloux, 2012; Brown et al., 2017). Kratom is currently consumed for much the same reasons and increasingly to ameliorate withdrawal from other substances (opioids, alcohol, and amphetamines) (Saingam et al., 2013; Singh et al., 2017; Singh et al., 2020; Singh et al., 2025). In the United States (US), freshly harvested leaf is not readily available so dried leaf is imported from Southeast Asia and used in a wide variety of product formulations. In the past decade, the kratom marketplace has changed–what once was just dried leaf material now includes extracts, gummies, tablets, capsules, tea bags, liquid shots, sublingual, and other edible formulations.

Self-report surveys from US consumers indicate that kratom is being used for the self-treatment of pain, mental health conditions, mitigation of substance use withdrawal symptoms, and for overall wellness (Garcia-Romeu et al., 2020; Smith et al., 2021; Smith et al., 2022c; Grundmann et al., 2023a; Smith et al., 2024; Swogger et al., 2026). These surveys report few serious adverse events, but with a changing product market, there has been an increase in reports to Poison Centers and kratom regulation (Towers et al., 2026). Exposures and case-reports often fail to identify the specific kratom product or product type, making causation between kratom use and adverse outcomes difficult to prove (Smith et al., 2022a). Further, it has been demonstrated that many products labelled as kratom are adulterated with undeclared substances and in some cases are not kratom at all (Lydecker et al., 2016; Nacca et al., 2020; LeSaint et al., 2022; Brown et al., 2026). Explicitly defining what is, and perhaps more importantly, is not kratom is paramount. This document is meant to define and delineate different kratom products and will provide evidence-based guidance for local, state, and federal governments regarding regulation of kratom and kratom-derived products.

Kratom is the common name used for referring to both the tree, *M. speciosa* and the leaves which across Southeast Asia are also referred to as biak or ketum (Malaysia), keton (Indonesia), beinsa (Myanmar), mambog or lugub (Phillipines), ithang, thom, or kratom (Thailand), and giam (Vietnam) (Raffa, 2015). The native kratom leaf contains a multitude of plant secondary metabolites including alkaloids, flavonoids, polyphenolic compounds, triterpenoids, saponins, monoterpenes, and secoiridoids (Brown et al., 2017). Alkaloids are recognized as the primary active compounds in kratom leaves, representing 0.5%–3.0% of the total weight (Hassan et al., 2013; Kruegel and Grundmann, 2018; Sengnon et al., 2023). The most abundant alkaloid is mitragynine and is reported to comprise 51%–82% of the total alkaloid fraction. On a percent weight basis, mitragynine ranges from <1.0% but usually does not exceed 2.5% of the total leaf weight (Hassan et al., 2013; Mudge and Brown, 2017; Kruegel and Grundmann, 2018; Sharma et al., 2019; Todd et al., 2020; Sengnon et al., 2023). Other major alkaloids found within the leaf material include paynantheine, speciogynine and speciociliatine (Sharma et al., 2019; Chear et al., 2021; Manwill et al., 2022; Sengnon et al., 2023).

Mitragynine is metabolized in the human body to some degree into 7-hydroxymitragynine (7-HMG), a transient, phase 1 metabolic byproduct (Kamble et al., 2019; Mongar et al., 2024). Although initially thought to be present in native kratom leaf material, it is now recognized as an oxidative byproduct of mitragynine and has been observed only in some post-harvest kratom leaf and kratom products at trace levels (0.003%–0.04% w/w) (Chear et al., 2021; Zhang et al., 2022; Karunakaran et al., 2024; Brown et al., 2026). The pharmacology of 7-HMG drastically differs from mitragynine and the other natural kratom alkaloids, exerting effects similar to conventional opioids (Matsumoto et al., 2006; Hemby et al., 2019; Hiranita et al., 2020; Obeng et al., 2021; Berthold et al., 2022). A recent study has reported 7-HMG induced significant respiratory depression comparable to morphine, which was reversed by naloxone, while mitragynine was found to increase respiratory frequency and was unaffected by naloxone (Zuarth Gonzalez et al., 2025). These findings highlight the critical differences between the naturally occurring kratom alkaloid mitragynine, and the metabolite 7-HMG. In late 2023, products with semi-synthetically produced 7-HMG were introduced into the marketplace with high percentages of 7-HMG by weight–much higher than could be obtained from nature.

From a regulatory perspective in the United States, kratom leaf material is a botanical that qualifies as a new dietary ingredient under section 201(ff)(1) of the Federal Food, Drug, and Cosmetic Act (FD&C Act) [21 U.S.C 323(ff) (1)]. From a consumer products perspective, kratom is used as a broad term referring to dried leaf material, extracts, and concentrates sold in a variety of forms including powders, capsules/tablets/pills, tinctures, beverages, shots, gummies, and tea bags. Herein, kratom and kratom-derived products will be classified and described, starting with kratom leaf material and moving stepwise away from the botanical to products that are not kratom and must be labelled and regulated in a manner that better reflects these products.

### 1.1 Defining kratom and kratom-derived products in the United States

Kratom as a consumer product refers to the leaves (fresh or dried) of *M. speciosa* which may be cut, crushed, ground, or pulverized. Mitragynine levels should range from 0.5%–2.5% by total dried leaf weight. The three other major alkaloids, speciociliatine, paynantheine and speciogynine comprise 0.1%–1.0% of leaf material by dry weight. Together, these four alkaloids can be used to define the ‘total alkaloid content’ of kratom, which should be <3.0% by weight. To date there have been more than 40 alkaloids identified in kratom leaf, however the vast majority are at trace levels. Outside of the four aforementioned major alkaloids, no other alkaloid should make up > 0.1% by dry leaf weight. Further, no degradation products, including 7-hydroxymitragynine, should be present >0.01% by weight.

Any product or material that is outside the definition of native kratom leaf material, as stated above, constitutes a kratom-derived product and may fall into the following broad extract categories: traditional botanical preparations (Category 1), concentrated extracts (Category 2), isolates, which may reflect the natural compositional ratio of alkaloids (Category 3a) or isolated fractions or individual alkaloids that are not representative of the natural kratom alkaloid distribution (Category 3b) and Category 4, novel drug substances. Which are not kratom and includes synthetic duplicates of kratom alkaloids and chemically transformed or altered alkaloids not found naturally in kratom leaf.

Traditional botanical preparations (Category 1) may include aqueous preparations with or without biomass, such as tea or decoctions, or fluidized (typically by aqueous ethanol) extracts with <10% alkaloid content. It should be noted that extracts fluidized using non-traditional solvents and concentrated extracts (Category 2) fall outside any traditional or historical preparation and as such should be considered as unique new dietary ingredients. Concentrated extracts (Category 2) may include semi-purified (10%–40% alkaloid content) and purified (40%–60% alkaloid content) materials. Isolates (Category 3) that are enriched to the point of only containing the alkaloid fraction (>60% alkaloid content) and are devoid of other plant phytochemicals, may be considered as an extract and qualify as a new dietary ingredient if effort has been made to conserve the natural alkaloid compositional ratios (Category 3a) and it has been demonstrated that no chemical alteration has occurred. Kratom, traditional botanical preparations of kratom, and kratom-derived extracts (Category 1, 2 and potentially 3a) qualify as dietary ingredients under section 201(ff)(1) of the Federal Food, Drug, and Cosmetic Act (the Act) [21 U.S.C. 321(ff) (1)] (See Figure 1). Products containing dietary ingredients are dietary supplements and as such are limited to products that are intended for ingestion in tablet, capsule, powder, softgel, gelcap, liquid, or other form, that are not represented as conventional food and that are labeled and regulated as dietary supplements.

**FIGURE 1:**
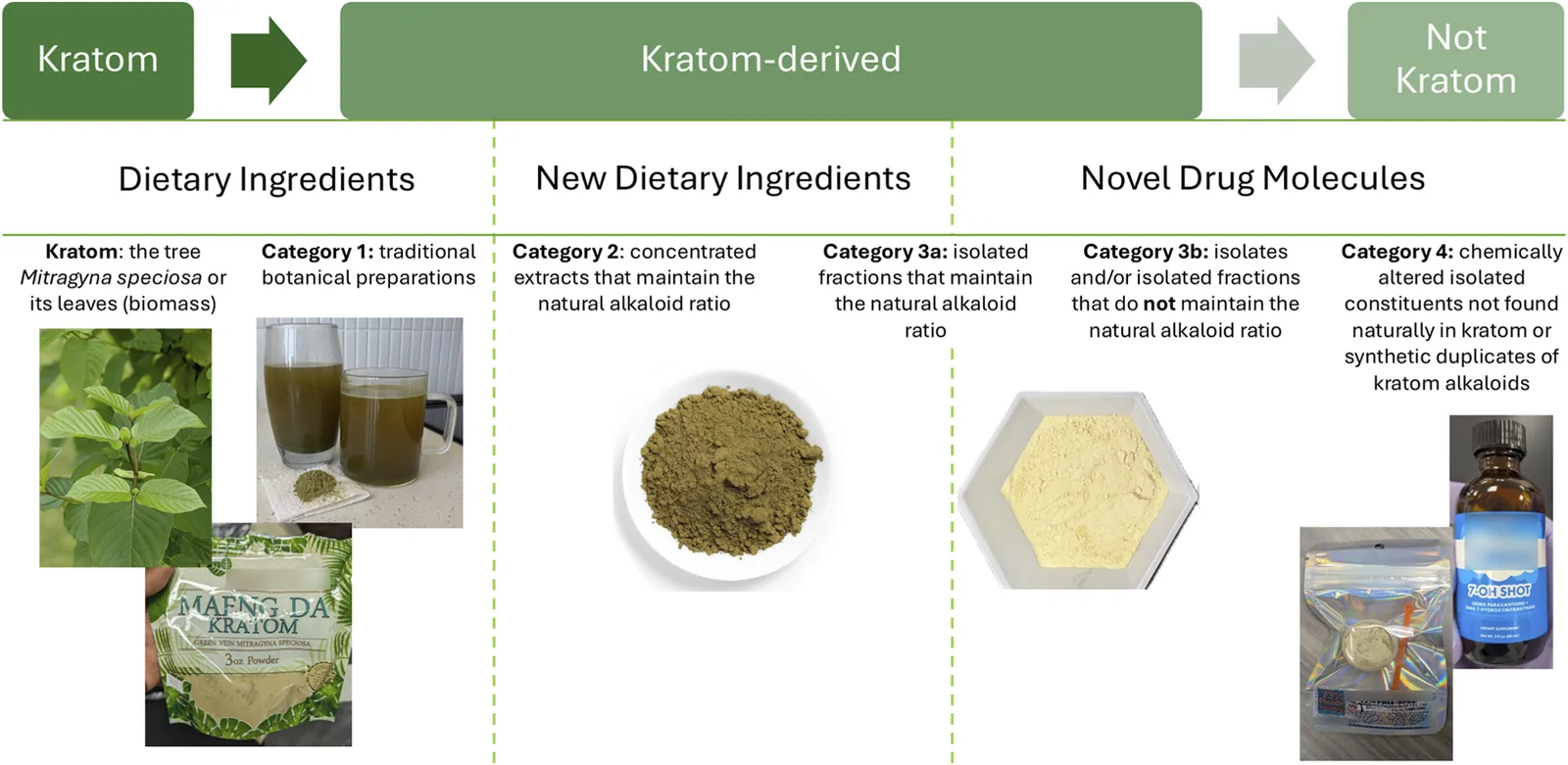
Kratom definitions and designations within the United States regulatory framework.

For Category 3b, isolated alkaloid fractions or compounds that do not reflect the natural composition of kratom, do not necessarily qualify as dietary ingredients and we are recommending they be treated as novel drugs. While there is precedent for plant derived molecules to be categorized as dietary ingredients, such as resveratrol, naturally found in the diet from grapes and peanuts, and (−)-epigallocatechin-3-gallate (EGCG) derived from green tea, there is also a history of safe use that accompanies these components. Unlike components that are pervasive through the food supply, the isolated, pure alkaloids of kratom do not have an extensive history of safe use in that format. Synthetic versions of botanical constituents may only be used in a dietary supplement if it qualifies as a “dietary substance,” i.e., a substance commonly used in conventional foods, such as synthetic vanilla, cinnamon, caffeine and citric acid (See FDA Guidance on Dietary Supplements: New Dietary Ingredient Notification and Related Issues, 2016). As such synthetic duplicates of natural alkaloids are grouped under novel drug substances (Category 4) along with the chemically altered alkaloids that are synthetically derived, such as 7-hydroxymitragynine and mitragynine pseudoindoxyl. Isolates containing one or more purified alkaloids that are not consistent with natural alkaloid ratios (Category 3b), synthetic duplicates of natural alkaloids, and chemically altered derivatives of alkaloids (Category 4) are defined as novel drug products and should be regulated as such (See Figure 1).

Recently, the marketplace has been flooded with products that can at best be called “kratom-inspired”. These products include isolated constituents that have been chemically altered (i.e., semi-synthetic oxidation of mitragynine into 7-hydroxymitragynine or similar processes) or synthetic alkaloids derived from 7-hydroxymitragynine or any other kratom alkaloid (Alsbrook et al., 2025; Hill et al., 2025; Brown et al., 2026; Sheehan et al., 2026). These constitute category 4 defined as novel drug molecules and should be regulated as such.

This categorization of products is largely based on the regulatory framework in the United States, as such the approach to appropriate regulatory action may differ in other countries. One such example is the Czech Republic which on 9 October 2024, approved an amendment to their Addictive Substances Act which introduced a differentiation of three groups of substances: a) addictive substances, b) psychomodulatory substances and c) psychoactive substances. Kratom (Group 1) and kratom extracts (Group 2, 3a) were classified as psychomodulatory substances, which were defined in the Amendment as substances that do not pose a serious risk to public health or a risk of serious social impacts on individuals and society (Mravcik et al., 2024). The Amendment also introduced requirements concerning the handling of psychomodulatory substances, including their research, manufacture, export, import, purchase and placement on the market (Dékány et al., 2025).

Regulatory policy should be built around these definitions and the safety data available for each category.

## 2 Policy assessment

### 2.1 Current regulatory landscape

Kratom first received federal regulatory action in 2012 when the US Food and Drug Administration (FDA) issued an import alert referring to kratom as an unapproved drug; this was changed in 2013 in the issuance of Import Alert 54–15 which describes kratom as a dietary ingredient under section 201(ff)(1) of the Federal Food, Drug, and Cosmetic Act (the Act) [21 U.S.C. 321(ff) (1)]. FDA further stated that when marketed as a dietary ingredient, they consider kratom to be a new dietary ingredient under section 413(d) of the Act [21 U.S.C. 350b(d)] because, to the best of the agency’s knowledge, there is no information demonstrating that this substance was marketed as a dietary ingredient in the United States before 15 October 1994.

Kratom faced further scrutiny on 30 August 2016, when the US Drug Enforcement Administration (DEA) announced its intention to place the active materials (mitragynine and 7-HMG) from kratom to Schedule I of the Controlled Substances Act (CSA) (DEA, 2026). This was later rescinded, but kratom and kratom-derived product legality continues to be debated. The FDA website states that ‘kratom is not lawfully marketed in the US as a drug product, a dietary supplement, or a food additive in conventional food’ (FDA, 2025a). But in July 2025, the FDA made recommendations to restrict 7-hydroxymitragynine products without limiting or scheduling kratom leaf products (FDA, 2025b). The agency recommended to the DEA that 7-HMG be placed in Schedule I of the CSA, but no federal action from the DEA has been taken on the date of this publication.

In the interim, states and local municipalities have passed regulations regarding kratom and kratom-derived products that vary widely in content. As of July 2026, there are nine states with an outright ban (Alabama, Arkansas, Connecticut, Indiana, Kansas, Louisiana, Tennessee, Vermont, and Wisconsin), California has a *de facto* ban through actions of the Public Health Department, and five states are considering a full ban (Michigan, Ohio, Idaho, Illinois, Maryland). There are twenty states where kratom is legal with minimal restrictions (Alaska, Delaware, Hawaii, Iowa, Maine, Massachusetts, Minnesota, Missouri, Montana, Nebraska, New Hampshire, New Jersey, New Mexico, New York, North Carolina, North Dakota, Pennsylvania, South Dakota, Washington, and Wyoming) and in the final fifteen states kratom is legal with some statewide regulations surrounding its sale and use, often referred to as Kratom Consumer Protection Acts (Arizona, Colorado, Florida, Georgia, Kentucky, Mississippi, Nevada, Oklahoma, Oregon, Rhode Island, South Carolina, Texas, Utah, Virginia, and West Virginia). Some statewide legislation simply puts an age-gate on kratom purchase and use (18 or 21 years old) while others have limits on the alkaloid content and serving size restrictions, along with labeling requirements. In the absence of federal regulation of kratom products, differences in policy are to be expected between states, but these evidence-based guidelines for kratom and kratom-derived products will help prevent loopholes that would allow irresponsible manufacturers to thrive.

### 2.2 Regulatory options and implications

Broadly there are always three options available for botanical products: ban and/or control, regulation, or no regulatory action. Bans or inaction are always the easiest solutions, but often not the most desirable or helpful to current consumers of such products. Not enforcing regulations allow for the isolated (Category 3), and semi-synthetic and synthetic products (Category 4) to penetrate the market, which puts consumers at an unreasonable safety risk. These products have no history of safe-use or any peer reviewed scientific literature to support use in humans. An outright ban on kratom or Category 1 products also is unsupported. There is insufficient evidence to suggest that kratom or traditional botanical preparations should be banned and/or scheduled based on available pre-clinical, observational (survey), historical use in Southeast Asia, and human clinical trials. Since the first scheduling attempt of mitragynine a decade ago, no new data has presented itself to warrant such action. There is no peer reviewed evidence that properly labeled, tested, and used kratom presents risks equivalent to other Schedule I compounds. International bodies, like the World Health Organization (WHO), have recognized kratom as a natural botanical of which there is insufficient evidence for a scheduling recommendation (WHO, 2021).

## 3 Actionable recommendations

### 3.1 Manufacturing

All manufacturers of kratom or kratom-derived products must follow the relevant regulatory requirements. This includes the Dietary Supplement Health and Education Act of 1994 (DSHEA), the Dietary Supplement and Nonprescription Drug Consumer Protection Act, and the regulatory sections of the Food, Drug, and Cosmetics Act on Food Labeling, Adulterated Foods, and Misbranded Foods. Manufacturers must follow the Code of Federal Regulations 21 CFR Part 111 Current Good Manufacturing Practice (cGMP) in Manufacturing, Packaging, Labeling, or Holding Operations for Dietary Supplements.

The cGMP regulations require that manufacturers identify the steps in their process(es) that require control and establish written specifications for identity, purity, strength, and composition, including limits on contaminants that may lead to adulteration of the finished product. Furthermore, Section 111.75 of the cGMP final rule compels manufacturers to verify that these specifications have been met through appropriate, scientifically valid testing or examination. Written specifications, test methods, demonstration of suitability and validity of test methods, master manufacturing records and batch records are all required, as is the collection of adverse event reports.

Quality specifications, including levels and profiles of desirable and undesirable constituents, are created for a variety of reasons. Specific chemical constituents, parameters, and/or profiles can be set to define product properties such as permissible natural toxin and pesticide levels and microbial load (and by proxy, safety), phytochemical marker levels (and by proxy, strength or content), ingredient identity, and provenance. It is the responsibility of the manufacturer to ensure they set specifications for any reasonably anticipated deleterious substance.

Additionally, it is recommended that all kratom leaf, Category 1 (traditional preparations), and Category 2 (concentrated extracts) products have clear and obvious serving measurements. Packages for solid dosages should limit the doses available by using blister packaging or packaging that will limit the number of servings in a single package. Packages for liquid dosing forms must include a measured device to allow individuals to consume the accurate indicated serving size.

### 3.2 Labeling

All labeling must meet the provisions in 21 CFR Part 111. Supplement labels are similar to food labels. They must have a principal display panel (PDP) and an information panel. The PDP must bear a statement of identity with the words ‘‘Dietary Supplement’’, ‘‘Nutritional Supplement’’, or ‘‘ Supplement’’ and a net quantity. The information panel must include the name and address of the manufacturer, distributor, or packager along with a telephone number for reporting adverse events.

Dietary supplement labels must have a supplement facts panel that includes the amount of each dietary ingredient (the purported bioactive) per serving along with the percent daily value (DV) provided by this amount of ingredient. If no DV has been established for the ingredient, as is the case for kratom and kratom-derived products at this point, an asterisk must be provided in the DV column with the legend “no daily value established” at the bottom of the panel. Below the supplement facts panel, all ingredients must be listed by common name in descending order of predominance. If the ingredient is a ‘proprietary blend’, the amount of the blend must be listed in the facts box, with the ingredients of the blend listed below in descending order of predominance in the blend.

Both health claims and statements of nutritional support (“structure-function” claims) are permitted on labels. Health claims must be nutrient related and pre-reviewed by the FDA. Unlike health claims, structure-function claims are not pre-approved by the FDA. The DSHEA requires the manufacturer to have substantiation data supporting that the claim is truthful and not misleading and must submit a notification with the text of the claim to the FDA no later than 30 days after marketing the dietary supplement with the claim. While the Act requires that manufacturers submit the text of the claim to FDA, it does not require that substantiation evidence be submitted to the agency. If a dietary supplement label includes a structure/function claim, it must state in a disclaimer that the FDA has not evaluated the claim. The disclaimer must also state that the dietary supplement product is not intended to “diagnose, treat, cure or prevent any disease”, as only drug products can legally make such a disease claim.

In line with cGMP, all kratom and kratom-derived product labels must include the word “kratom” in the ingredient list. Labels need to have a serving size recommendation, recommended servings per day, use instructions including the recommended duration of use, expiration date, a complete list of all ingredients, the % weight amount of mitragynine and lot number. Additionally, based on safety concerns it is suggested that all kratom and kratom-derived product labels identify the 7-hydroxymitragynine content per serving or the limit of detection of the method, if the product contains non-detectable levels.

Products should be labeled for sale to adults (over 21 years of age) and contain the following statements: not for use in pregnant or lactating individuals or those trying to become pregnant. Do not use if you have a medical condition or are taking any medications. Consult your healthcare provider before using this product. This product is meant to be consumed orally only. Do not exceed the recommended serving size or recommended servings per day. Kratom and kratom-derived products should also contain the following warnings: Do not take with drugs or consume with alcohol, to do so could cause serious injury including fatality. Use of this product may cause physical dependence. Discontinue use if you experience any adverse effects. The above information must be present on the label of the product. For small-label products it is recommended that innovative packaging such as an insert be used to ensure the information is present.

### 3.3 Serving size limitations

Based on the available scientific data (Assanangkornchai et al., 2007; Singh et al., 2014; Swogger et al., 2015; Grundmann, 2017; Smith and Lawson, 2017; Garcia-Romeu et al., 2020; Davidson et al., 2021; Henningfield et al., 2021; Smith et al., 2022b; Grundmann et al., 2023b; Coe et al., 2026) for kratom leaf products and traditional botanical preparations (Category 1), as described above, there is reasonable assurance that such products when properly prepared and used as directed are safe with the following recommended limits: a maximum mitragynine level per serving of 40 mg and recommended daily intake of no more than 2 servings in a 24 h period (totaling 80 mg of mitragynine in a 24 h period). Again, it must be stressed that this recommendation and the history of use is only applicable to products prepared as per tradition (kratom leaf products and Category 1).

There is insufficient scientific data to make definitive recommendations and statements for kratom-derived products in Category 2, which may include chemically altered materials. It is the responsibility of manufacturers who make extracts to demonstrate their process does not induce any chemical alteration. Further, any extracts, including fluidized extracts that are not a traditional kratom preparation, such as tinctures, require safety studies be conducted on the dietary ingredient.

However, in the absence of safety data, it is recommended that under the following conditions:

1. Product contains not more than 40 mg of mitragynine per serving,
2. Mitragynine is the most abundant alkaloid (>40% of total alkaloids),
3. 7-hydroxymitragynine is <0.01% of final product on a dry weight basis (w/w), it may be reasonably anticipated that the same recommendation as made for kratom leaf and Category 1 products may apply. However, it is additionally recommended that anyone selling extract material (Category 2, 3a), should demonstrate the product is not chemically altered, obtain safety data for the ingredient, and submit a new dietary ingredient notification as per the regulatory requirements. There is no scientific support or recommended serving sizes for the safe consumption of Category 3b products and Category 4 products which should be treated as novel, unapproved drugs with no basis for safety and should be regulated as drugs in the current regulatory framework in the United States.

## 4 Conclusion

Complex botanicals, like *Mitragyna speciosa*, often pose regulatory challenges driven by innovation and adulteration. These types of plants have been consumed for centuries, with few adverse side effects, but when introduced to the United States market, isolation of active compounds and chemical synthesis of novel chemical entities proliferate and corrupt the unaltered native botanical. Policy in the United States must find a delicate balance between protecting public health and also keeping available botanicals for responsible adult consumers who choose them as part of their overall wellness. The proposed regulatory framework is foremost based on the definition of a dietary supplement and a new drug compound by United States agencies and thus limits the applicability to other countries which may differ in policy and regulation. Furthermore, it must be acknowledged that legislative frameworks are shaped by cultural and societal norms which are specific to countries or even regionss. Within the US framework, banning or enacting strict scheduling on kratom leaf and Category 1 products does not agree with the tradition of use and scientific literature. This is not to say there is no risk with kratom consumption–there very clearly is. But that risk increases with lack of education, irresponsible manufacturing practices, false marketing and advertising of products that are substantially different in their composition from native kratom products, and lack of serving size recommendations. Implementing these simple, clear guidelines for kratom and kratom-derived products will help to build regulation that supports responsible use, provides clear instructions to consumers, and recommends limitations on alkaloid levels per serving and total daily alkaloid intake.

## Data Availability

The original contributions presented in the study are included in the article/supplementary material, further inquiries can be directed to the corresponding authors.
